# Association between hypovitaminosis D and uterine leiomyomas among women of reproductive age attending selected hospitals in Uganda: A multicenter cross-sectional study

**DOI:** 10.1371/journal.pone.0354584

**Published:** 2026-09-08

**Authors:** Senaji Khamonya Nightingale, Rogers Kajabwangu, Maxwell Okello, Emmanuel Okurut, Marie Pascaline Sabine Ishimwe, Peter Okello, Happy Tukirinawe, Perez Fernando, Ubarnel Almenares, James Okello, Yarine Fajardo, Theoneste Hakizimana

**Affiliations:** 1 Department of Obstetrics and Gynecology, Kampala International University, Ishaka, Uganda; 2 Department of Obstetrics and Gynecology, Mbarara University of Science and Technology, Mbarara, Uganda; 3 Department of Pediatrics and Child Health, Kampala International University, Ishaka, Uganda; Peking University People’s Hospital, CHINA

## Abstract

**Background:**

Uterine leiomyoma, is a common benign neoplasm among women of reproductive age with a potential of causing significant health complications and financial burdens in severe cases. Recent research suggests a possible link between vitamin D deficiency and uterine leiomyoma development; however, this relationship has not been established in the Ugandan population. For this reason, there is no individualized or public health interventions targeting this modifiable risk factor. The study aimed to determine the relationship between hypovitaminosis D and uterine leiomyoma among women of reproductive age attending Jinja, Lira and Fort Portal Regional Referral Hospitals in Uganda.

**Methods:**

A cross-sectional multicentre study was conducted at Jinja, Lira and Fort Portal Regional Referral Hospitals between 1^st^ October 2022–31^st^ January 2023. 246 non-pregnant women of reproductive age were included in the study. Questionnaires were administered to the participants, their serum vitamin D analysed and a transabdominal pelvic scan done. A p value of ≤0.05 was the threshold for statistical significance.

**Results:**

The prevalence of hypovitaminosis D among participants was high at 54.1%. There was no significant difference in the proportions of leiomyoma among those with and without hypovitaminosis D (χ^2^ = 0.503, p = 0.478 > 0.05). The mean serum vitamin D level was lower in individuals with uterine leiomyomas compared to those without (19.954 ± 9.77 versus 21.552 ± 9.54 [95% CI: −0.04 to 4.24, p = 0.235]). There was no statistically significant association between hypovitaminosis D and uterine leiomyoma (OR 1.22, 95% CI 0.70–2.12; p = 0.478). There was a weak negative correlation observed between serum vitamin D levels and leiomyoma site number (r = −0.2482, p = 0.0342 < 0.05).

**Conclusion and recommendations:**

The prevalence of hypovitaminosis D was high among women of reproductive age. There was no statistically significant relationship between hypovitaminosis D and uterine leiomyomas despite a weak negative correlation between vitamin D levels and leiomyoma site number which was statistically significant. High prevalence of hypovitaminosis D underscores the need for health education and public health measures by healthcare providers to manage and prevent vitamin D deficiency-related morbidity among women of reproductive age. Further research by the scientists is needed to investigate the potential association between hypovitaminosis D and uterine leiomyoma, in regards to increased site number, among the African population.

## Introduction

About half of the world’s population has vitamin D deficiency, including 18.46% of Africans [1]. Sakyi et al., observed that despite the abundance of sunlight in Ghana, 43.6 percent of the adult Ghanaian population was vitamin D deficient [2]. The prevalence of hypovitaminosis D among Egyptian women of childbearing age was about 43% of the participants for Vitamin D deficient and 13% for Vitamin D insufficiency while another study done in Sudan found a prevalence of 82.6% for vitamin D deficiency among Sudanese women [3,4]. A majority of these women however use covered clothing style.

The prevalence of vitamin D deficiency in Uganda was estimated to be between 9–20% based on studies associating vitamin D deficiency among HIV patients with Tuberculosis co-infection and another study associating it with acute stroke [5]. The Conesa-Botella et al study population included male participants with a median age of 35 years and the subsequent study by Kiggundu et al included participants with a median age of 69 years, both males and females, with the proportion of females being 54 percent. There was no study done to assess the prevalence of vitamin D deficiency among non-pregnant women of reproductive age in Uganda [5,6]

It has also been posited that abundant melanin on black skin absorbs UVB rays decreasing vitamin D synthesis by approximately 99% [7]. This observation indicates that sufficient sunshine alone may not be the only determinant for vitamin D sufficiency in the tropics. It is also pertinent to mention that most of the studies on uterine leiomyoma have included the African American women as participants whom despite similarities with the native Africans, are exposed to different climatic conditions when compared to the tropical, semi-arid and arid climates that are dominant in Africa with more hours of sunshine during the course of the year.

However, despite the notable difference in geographic location between African Americans and Africans, studies done in Africa investigating the relationship between Vitamin D deficiency and uterine leiomyomas are consistent with the studies done in Turkey and India [8–10]. The sociodemographic characteristics of an individual can have a great impact on their vitamin D level. With urbanisation, women tend to spend more time indoors on the other hand Muslim women tend to cover most parts of their bodies with less skin exposure to the sun.

Uterine leiomyomas are common among women of reproductive age although their burden across populations is variable. Rates range from 4.5 to 68.6% [11]. By the time of onset of menopause uterine leiomyomas have been posited to occur in over 70% of women [12]. Between 20 and 25 percent of women, or over 235 million women, or 6.6 percent of all women worldwide, are thought to be affected by leiomyomas, according to 2010 World Health Organization research [13]. Among African studies, the reported prevalence of uterine leiomyomas varies considerably. A sonographic study from Northern Nigeria reported a prevalence of 12.1% [14], while a Ghanaian study reported a prevalence of 36% among women undergoing pelvic ultrasonography [15]. In Kenya, robust population-based prevalence estimates remain scarce, although available literature suggests uterine fibroids are common among women of reproductive age [16]

In Uganda, the prevalence of uterine leiomyoma was high at 28.2% among women who attended Mbarara Regional Referral hospital from November 2018 to February 2019 [17]. The rates reported may be an underestimate because uterine leiomyoma tends to be asymptomatic in 25% of women and symptoms have an insidious onset and therefore remains undiagnosed [18].

Black race was consistently linked to an elevated risk of uterine leiomyomas according to a study by Stewart et al on assessment of risk variables for uterine leiomyomas [11]. Comparing African American women to Caucasian women, uterine leiomyomas were found to be twice as common or even quadruple as common [19]

Vitamin D deficiency was discovered to be a significant modifiable component in the development of uterine leiomyoma [20,21]. Those with uterine leiomyomas have been found to have lower levels than women without uterine leiomyomas according to studies done in Europe- Italy [22]; Asia- China [23], India [24] and Africa – Egypt [25] and Nigeria [10]. In the aforementioned studies, some but not all studies have associated hypovitaminosis D with increased number and size of leiomyoma. Ugandan-specific evidence is needed because the available local literature on vitamin D has historically focused on selected patient groups, such as adults with HIV/TB co-infection and patients with acute stroke, and therefore may not reflect vitamin D status among non-pregnant women of reproductive age [5,6]. Meanwhile, uterine fibroids are a clinically important condition in Uganda; a study at Mbarara Regional Referral Hospital found a prevalence of 28.2% among women attending gynecology clinic [17]. Beyond epidemiologic relevance, vitamin D has been proposed to influence leiomyoma biology through inhibition of cellular proliferation, promotion of apoptosis, suppression of extracellular matrix accumulation, and modulation of steroid-hormone signalling [6]. However, the strength and consistency of these associations remain uncertain across populations, supporting the need for context-specific data from Ugandan women. This study sought to identify the prevalence of hypovitaminosis D among women of reproductive age in Uganda and proceeded to determine the relationship between hypovitaminosis D and uterine leiomyoma with its sonographic characteristics which was the main objective for this study.

## Methods

### Study design and setting

This was a multicenter hospital-based cross-sectional study conducted at Jinja, Lira, and Fort Portal Regional Referral Hospitals from 1 October 2022–31 January 2023. The study determined the prevalence of hypovitaminosis D; compared the proportion of uterine leiomyomas among women with and without hypovitaminosis D. Lastly, determined the relationship between hypovitaminosis D and uterine leiomyoma in conjunction with leiomyoma characteristics among women of reproductive age attending selected hospitals in Uganda. This multi-centre study conducted at the government-funded regional referral hospitals of Jinja, Fort portal and Lira. Jinja Regional Referral Hospital (JRRH) is located in Jinja city, in Eastern Uganda. Fort Portal Regional Referral Hospital is located in Fort Portal city, Western Uganda. These two facilities attend to an average of 80 gynaecologic patients per month. The Lira Regional Referral Hospital which lies in Northern Uganda serves an average of 140 gynaecologic patients per month.

### Participants

The study participants were all non-pregnant women of reproductive age (15–49 years) who agreed to take part in the study attending Gynecology outpatient clinic at Jinja, Fort Portal and Lira Regional Referral Hospitals. Consecutive sampling technique was used in this study. Women who were excluded from this study included: lactating mothers or those who stopped lactating within the last 6 months of data collection period; patients who had a history of hysterectomy; vitamin D supplementation in the last 6 months prior to data collection; patients who reported chronic medical disorders (for example, hepatic or renal) known to affect vitamin D metabolism. Critically and mentally ill patients were also excluded from this study.

### Variables and data sources

The main exposure variable was hypovitaminosis D. After the informed-consent process, participants who agreed to take part in the study completed a questionnaire capturing socio-demographic characteristics. A physical examination was then performed on all participants.

For quantitative serum vitamin D analysis, 2.5 mL of venous blood was collected aseptically from the cubital fossa of the non-dominant arm. Samples were transported immediately to the laboratory with a duly completed laboratory request form, separated, aliquoted into cryotubes, protected from light, and stored at −20°C until analysis using the ichroma™ vitamin D assay. Serum 25-hydroxyvitamin D levels were measured quantitatively and analyzed in this study primarily in two ways. **First, vitamin D was analyzed as a categorical exposure variable, with hypovitaminosis D defined as serum 25-hydroxyvitamin D < 20 ng/mL and sufficient vitamin D status defined as levels ≥20 ng/mL, in accordance with Institute of Medicine criteria** [26]. Second, serum vitamin D was analyzed as a continuous variable in relation to uterine leiomyoma and its sonographic characteristics. For operational description, vitamin D status was classified as deficiency (<12 ng/mL), insufficiency (12 to <20 ng/mL), and sufficiency (≥20 ng/mL), although the primary categorical analysis in this study used the broader hypovitaminosis D threshold of <20 ng/mL.

Pelvic transabdominal ultrasonography for the diagnosis of uterine leiomyoma, the dependent variable, was performed by experienced radiologists and sonographers (two per participating center) who had longstanding involvement in gynecological sonography. A GE Logiq P5 ultrasound machine with a 3.5–5 MHz curvilinear probe was used. Leiomyomas were defined sonographically as well-defined, symmetrical, hypoechoic, and heterogeneous masses. Leiomyoma characteristics, including location, number, size, and site according to FIGO classification, were identified and recorded on the ultrasonography request form.

For this study, “leiomyoma number” referred to the total count of distinct fibroids identified in a participant. “Leiomyoma location” referred to the anatomic compartment in which a leiomyoma was found, such as submucosal, intramural, subserosal, cervical, or hybrid. “Leiomyoma site” referred to the FIGO site classification based on the relationship of the leiomyoma to the endometrium and serosa. “More than one location” indicated fibroids occurring in more than one anatomic compartment, whereas “more than one site” indicated fibroids involving more than one FIGO site category.

### Sample size and power

Assuming a 95% confidence interval and a prevalence of 20% for vitamin D deficiency in Uganda [5], a sample size of 246 non-pregnant women of reproductive age was sufficient to assess the relationship between hypovitaminosis D and uterine leiomyomas.

### Data management and analysis

Data were entered, cleaned, and analyzed using the Statistical Package for the Social Sciences (SPSS). Descriptive statistics were used to summarize participant characteristics and key study variables. Continuous variables were summarized using means and standard deviations, while categorical variables were summarized using frequencies and percentages.

The prevalence of hypovitaminosis D was calculated as the proportion of women with serum 25-hydroxyvitamin D_3_ levels below 20 ng/mL among all enrolled participants and expressed as a percentage. This was presented graphically using a pie chart.

To compare the proportion of uterine leiomyomas among women with and without hypovitaminosis D, proportions were calculated as the number of participants with leiomyomas divided by the total number of participants in each vitamin D category. Group differences were assessed using the chi-square test. Statistical significance was defined as a p-value ≤0.05, with corresponding 95% confidence intervals.

To examine the association between hypovitaminosis D and uterine leiomyoma, bivariable logistic regression was first performed to estimate crude odds ratios (cORs) and 95% confidence intervals. Variables considered clinically relevant and/or associated with uterine leiomyoma at bivariable analysis were entered into a multivariable logistic regression model to adjust for potential confounding. Adjusted odds ratios (aORs) with 95% confidence intervals were reported.

Effect size for categorical associations was assessed using the Phi (φ) coefficient. Serum 25-hydroxyvitamin D_3_ levels were analyzed both as a continuous and a categorical variable. As a continuous variable, mean vitamin D levels were compared between women with and without uterine leiomyomas, and differences were assessed for statistical significance. As a categorical variable, hypovitaminosis D was defined as serum vitamin D levels <20 ng/mL, while sufficient vitamin D status was defined as levels ≥20 ng/mL, in accordance with Institute of Medicine criteria.

Pearson correlation analysis was performed to assess the relationship between continuous serum vitamin D levels and uterine leiomyoma sonographic characteristics, including leiomyoma number, site number, largest leiomyoma volume, and uterine corpus volume. Scatter plots were generated with individual data points to display the underlying data distribution. A least-squares linear regression line with 95% confidence intervals was superimposed to illustrate the direction and strength of associations. This approach enabled visual assessment of variability and linear trends between vitamin D levels and leiomyoma characteristics.

All statistically significant results were defined as those with p-values ≤0.05 and corresponding 95% confidence intervals. Results were presented in tables and figures as appropriate

### Ethical approval and consent to participate

This study was approved by the research ethics committee of Kampala International University, Western Campus (KIU-REC) with an approval number of KIU-2022–136. The study was registered with the Uganda National Council for Science and Technology (UNCST). All participants provided written informed consent. We followed the ethical standards for the regulation of research in humans in accordance with the Declaration of Helsinki.

## Results

### Characteristics of study participants

Of the 246 participants, the majority were Christians (90.2%), Langi, from rural area and had a monthly income of less than 100,000 Ugandan shillings (64.6%). Most of the participants did not consume alcohol (87.0%). Basoga tribe 36 out of 47 (76.6%) and rural residence were most affected. No major differences in vitamin D status across age groups, marital status, occupation, education level, religious affiliation, monthly income, or alcohol consumption (Table 1).

**Table 1:** Sociodemographic characteristics of study participants.

Variables	Hypovitaminosis D (<20 ng/mL)		
	No N = 113(45.9), n (%)	Yes N = 133(54.1), n (%)	Total N = 246, n (%)
Ethnicity			
Langi	43(38.1)	36(27.1)	79(32.1)
Busoga	11(9.7)	36(27.1)	47(19.1)
Batooro	33(29.2)	22(16.5)	55(22.4)
Others	26(23.0)	39(29.3)	65(26.4)
Residence			
Rural	72(63.7)	92(69.2)	164(66.7)
Urban	41(36.3)	41(30.8)	82(33.3)
Age			
<20	3(2.7)	2(1.5)	5(2.0)
20-29	40(35.4)	40(30.1)	80(32.5)
30-39	40(35.4)	55(41.4)	95(38.6)
40-49	30(26.6)	36(27.1)	66(26.8)
Marital status			
Married	74(65.5)	92(69.2)	166(67.5)
Single	39(34.5)	41(30.8)	80(32.5)
Occupation			
Unemployed	49(43.4)	54(40.6)	103(41.9)
Informal	43(38.1)	57(42.9)	100(40.7)
Formal	21(18.6)	22(16.5)	43(17.5)
Level of education			
Uneducated	9(8.0)	18(13.5)	27(11.0)
Primary	51(45.1)	53(39.9)	104(42.3)
Secondary	28(24.8)	39(29.3)	67(27.2)
Tertiary	25(22.1)	23(17.3)	48(19.5)
Religion			
Christian	104(92.0)	118(88.7)	222(90.2)
Muslim	8(7.1)	14(10.5)	22(8.9)
Others	1(0.9)	1(0.8)	2(0.8)
Monthly income (Ugandan shillings)			
<100000	73(64.6)	86(64.7)	159(64.6)
100000-500000	40(35.4)	47(35.3)	87(35.4)
Alcohol consumption			
Yes	15(13.3)	17(12.8)	32(13.0)
No	98(86.7)	116(87.2)	214(87.0)

### Prevalence of hypovitaminosis D among women of reproductive age attending selected hospitals in Uganda

**Fig 1:**
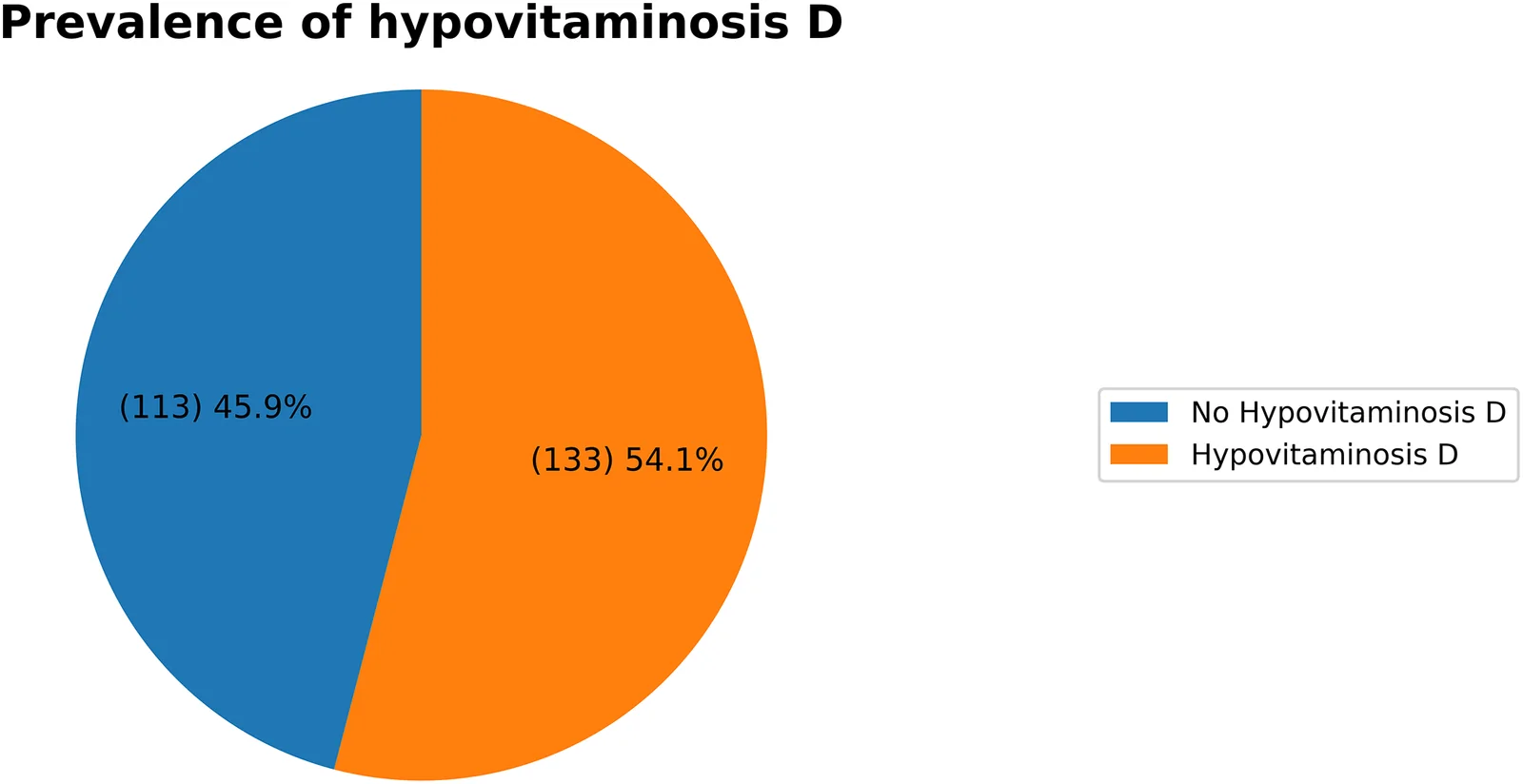
Prevalence of hypovitaminosis D among women of reproductive age attending selected hospitals in Uganda.

The prevalence of hypovitaminosis D was 54.1% (n = 133). However 45.9% (n = 113) did not have Hypovitaminosis D (Fig 1).

### Proportions comparison of uterine leiomyomas among study participants with and without hypovitaminosis D

Uterine leiomyoma was present in 29.7% (n = 73) and absent in 70.3% (n = 173) of the participants. Uterine leiomyoma was present in 31.6% and 27.4% among those with and without hypovitaminosis D respectively. There was no significant association between hypovitaminosis D and the presence of uterine leiomyomas in the sample (**χ**^**2**^0.503, p = 0.478 > 0.05) (Table 2).

**Table 2:** Proportions comparison of uterine leiomyomas among study participants.

Variables	Frequency	Leiomyomas		Chi-square (χ2)	p value
	N = 246, n (%)	No n = 173 (70.3), n (%)	Yes n = 73 (29.7), n (%)		
**Hypovitaminosis D**					
Absent	113(45.9)	82(72.6)	31(27.4)	0.503	0.478
Present	133(54.1)	91(68.4)	42(31.6)		

### Relationship between hypovitaminosis D and uterine leiomyoma

**Fig 2:**
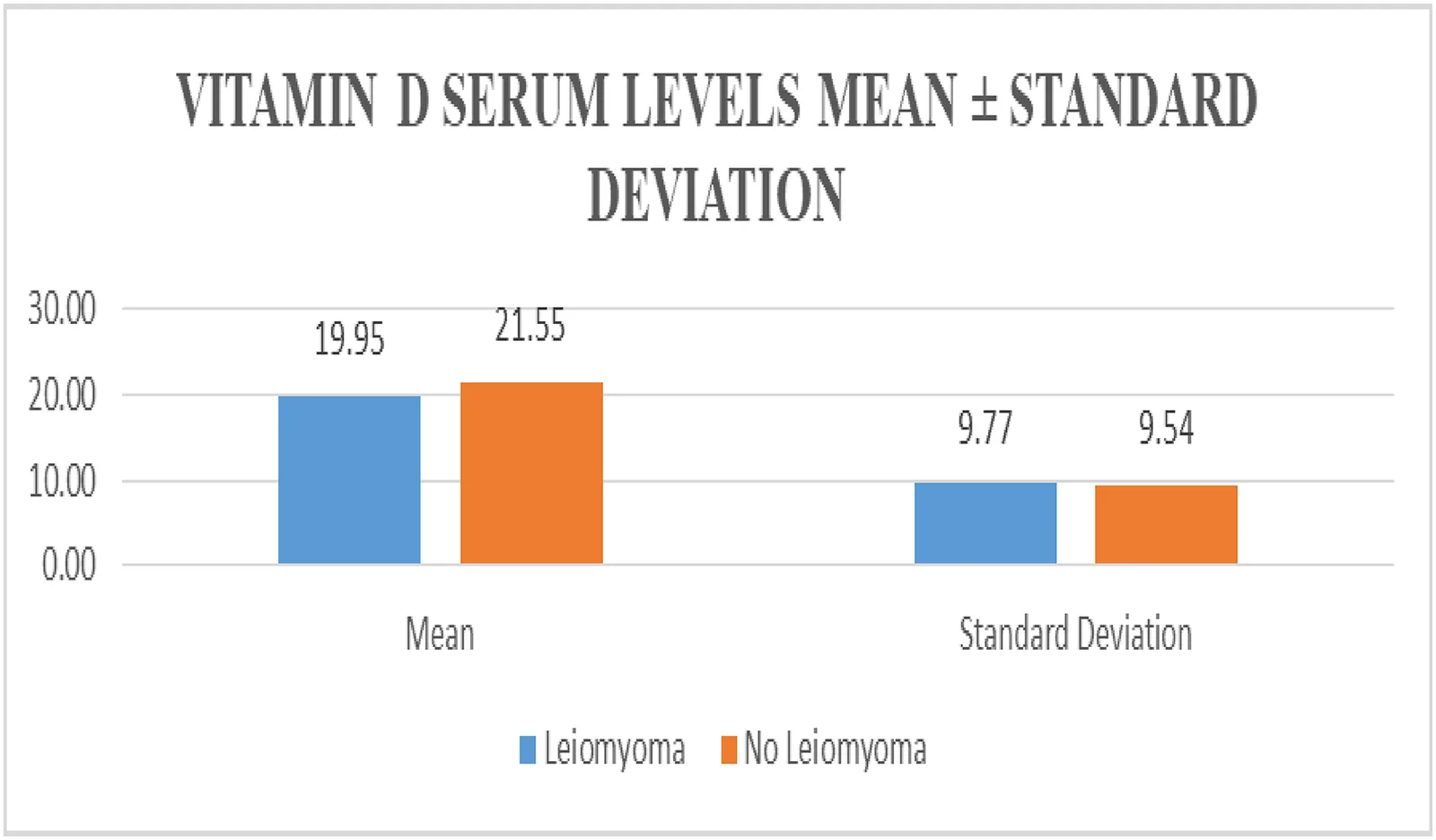
Comparison of mean serum vitamin D concentrations among women with and without uterine leiomyomas.

The mean serum vitamin D level was slightly lower in individuals with uterine leiomyomas compared to those without (19.954 ± 9.77 versus 21.552 ± 9.54 [95% CI: −0.04 to 4.24, p = 0.235]). The mean difference was 1.598 ng/mL. 54.1% had hypovitaminosis D and 45.9% did not (Fig 2). Among the total of 246 participants, 29.7% had leiomyoma, while 70.3% did not. Uterine leiomyoma was present in 42 of 133 women with hypovitaminosis D (31.6%) and in 31 of 113 women without hypovitaminosis D (27.4%). Women with hypovitaminosis D had higher unadjusted odds of uterine leiomyoma than women without hypovitaminosis D, but this association was not statistically significant (OR 1.496, 95% CI 0.448–4.991; p = 0.513) (Table 3).

**Table 3:** Relationship between Hypovitaminosis D and Uterine Leiomyoma.

Variables	Frequency	Hypovitaminosis D		cOR (95%CI)	P- Value	aOR(95% CI)	P-value
	N = 246, n (%)	No n = 113(45.9), n (%)	Yes n = 133(54.1), n (%)				
	**Leiomyomas**						
No	173(70.3)	82(72.6)	91(68.4)	Ref		Ref	
Yes	73(29.7)	31(27.4)	42(31.6)	1.22(0.70-2.12)	0.478	1.496(0.448-4.991)	0.513

cOR: Crude odds ratio, aOR: Adjusted odds ratio, CI: Confidence interval. Adjustment was done for age, parity, BMI, outdoor hours and physical activity.

### Relationship of hypovitaminosis D and uterine leiomyoma characteristics

Univariate analysis showed no statistically significant difference in the occurrence of hypovitaminosis D among women with leiomyoma, regardless of whether they had more than three leiomyomas (p = 0.422), more than one location (p = 0.665), or more than one site (p = 0.162). In bivariable logistic regression analysis, the odds of hypovitaminosis D were higher among women with more than three leiomyomas (OR 1.63, 95% CI: 0.49–5.35), more than one leiomyoma location (OR 1.24, 95% CI: 0.47–3.23), and more than one leiomyoma site (OR 2.11, 95% CI: 0.74–6.01), although none of these associations was statistically significant. Likewise, at multivariable level, there was no statistically significant association. Overall, hypovitaminosis D was not significantly associated with having more than three leiomyomas or more than one leiomyoma location. However, having more than one leiomyoma site showed a borderline trend toward a significant association with hypovitaminosis D (p = 0.065) (Table 4).

**Table 4:** Relationship of hypovitaminosis D and uterine leiomyoma characteristics.

Variables		Frequency	Hypovitaminosis D		cOR (95% CI)	p Value	aOR(95%CI)	p Value
		N = 73 n (%)	No (n %)	Yes (n%)				
More than 3 leiomyomas								
No		58(79.5)	26(44.8)	5(33.3)	Ref			
Yes		15(20.6)	32(55.2)	10(66.7)	1.63(0.49-5.35)	0.425	1.30(0.85-1.51)	0.254
More than 1 location								
No		45(61.64)	20(44.44)	25(55.56)	Ref			
Yes		28(38.36)	11(39.29)	17(60.71)	1.24(0.47-3.23)	0.665	1.16(0.76-1.79)	0.484
More than 1 site								
No		50(68.49)	24(48.00)	26(52.00)	Ref			
Yes		23(31.51)	7(30.43)	16(69.57)	2.11(0.74-6.01)	0.162	1.46(0.98-2.20)	0.065

cOR: Crude odds ratio, aOR: Adjusted odds ratio, CI: Confidence interval. Adjustment was done for age, parity, BMI, outdoor hours and physical activity.

### Relationship between vitamin D levels and uterine leiomyoma characteristics

**Fig 3:**
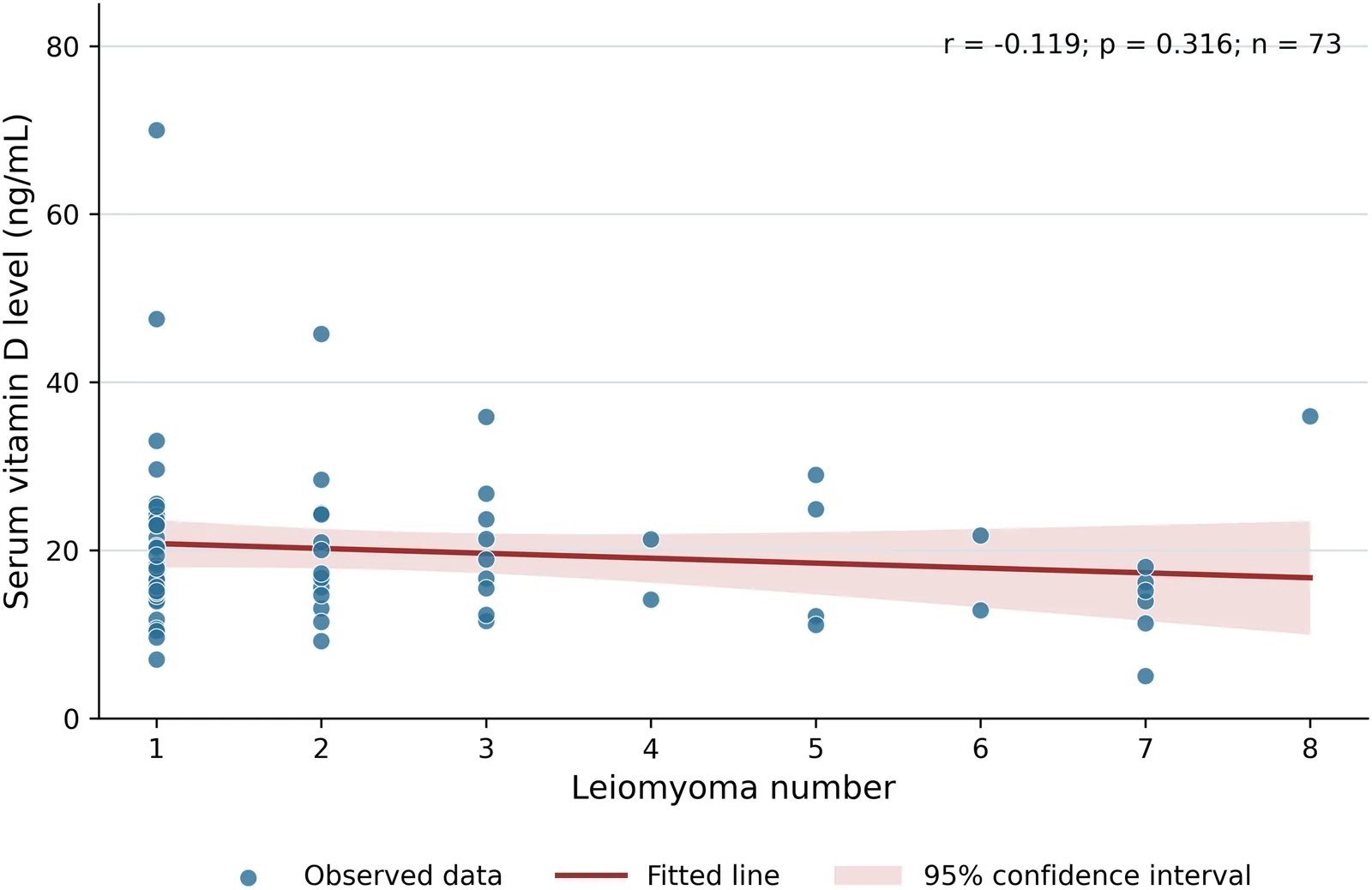
Scatter plot showing the relationship between serum vitamin D levels and leiomyoma number. Each dot represents an individual participant. The solid line represents the least-squares linear regression with 95% confidence intervals.

**Fig 4:**
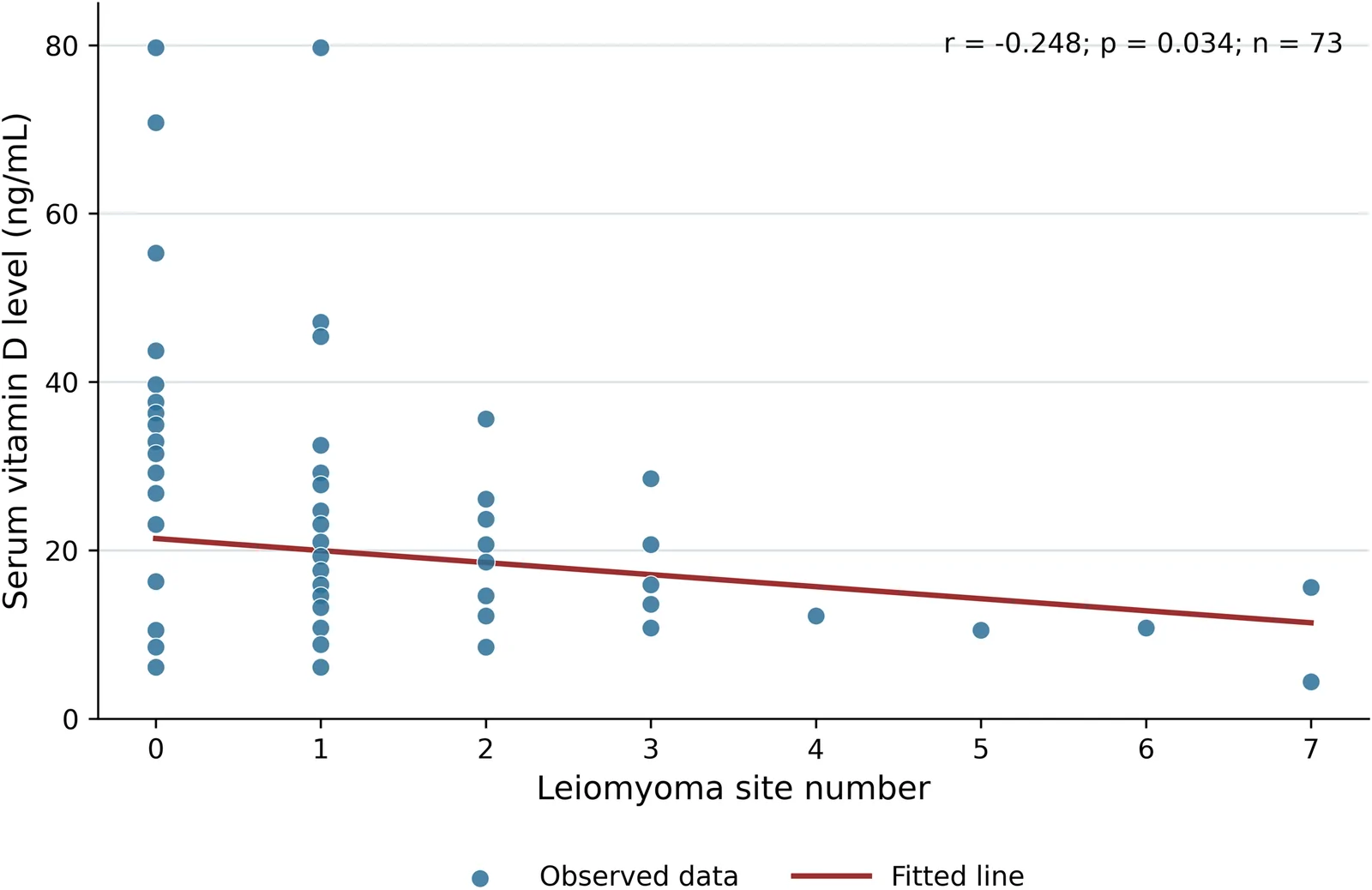
Scatter plot showing the relationship between serum vitamin D levels and leiomyoma site number. Individual data points are shown with a fitted linear regression line and 95% confidence intervals.

**Fig 5:**
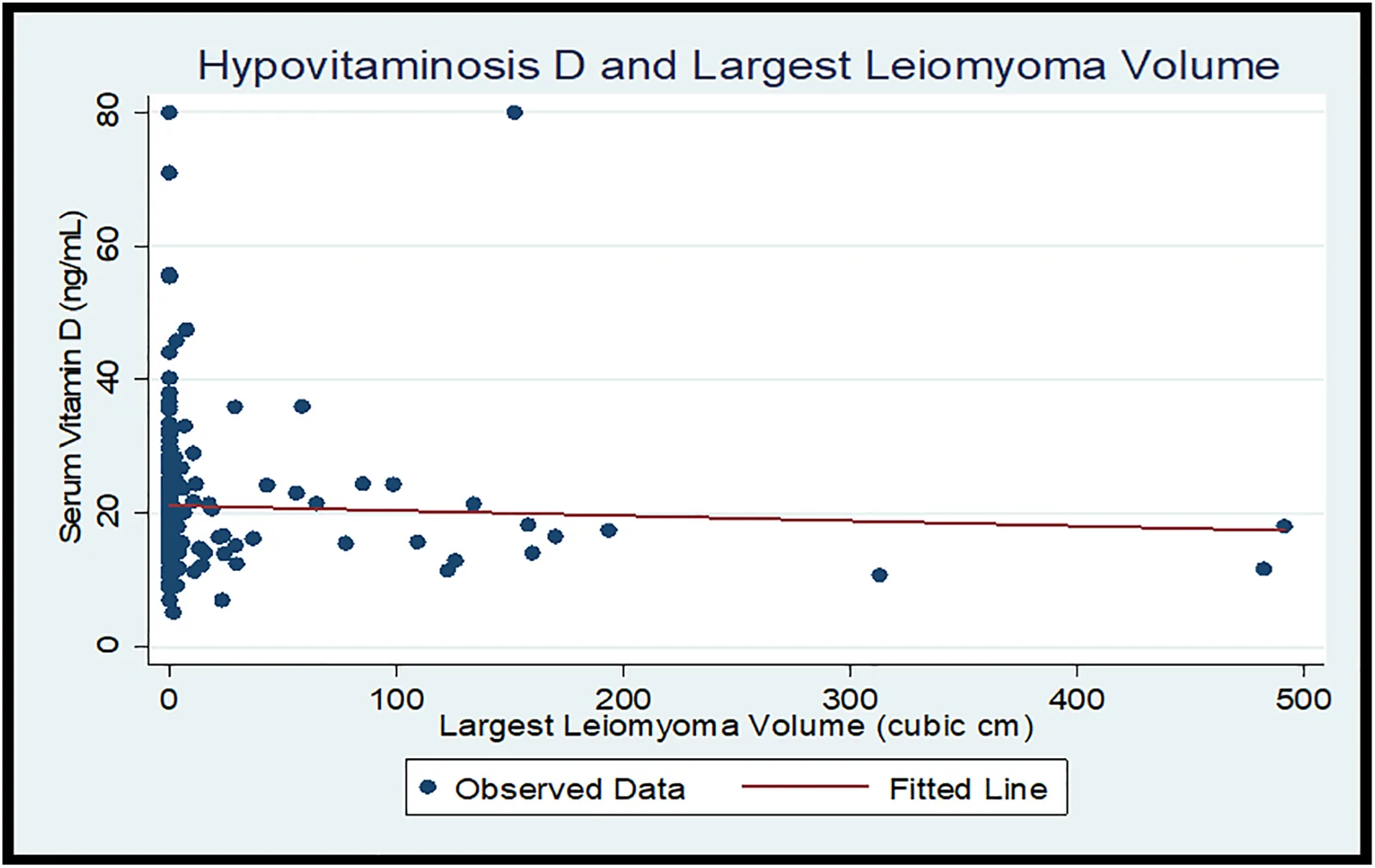
Scatter plot illustrating the association between serum vitamin D levels and largest leiomyoma volume, including a fitted regression line and 95% confidence intervals.

**Fig 6:**
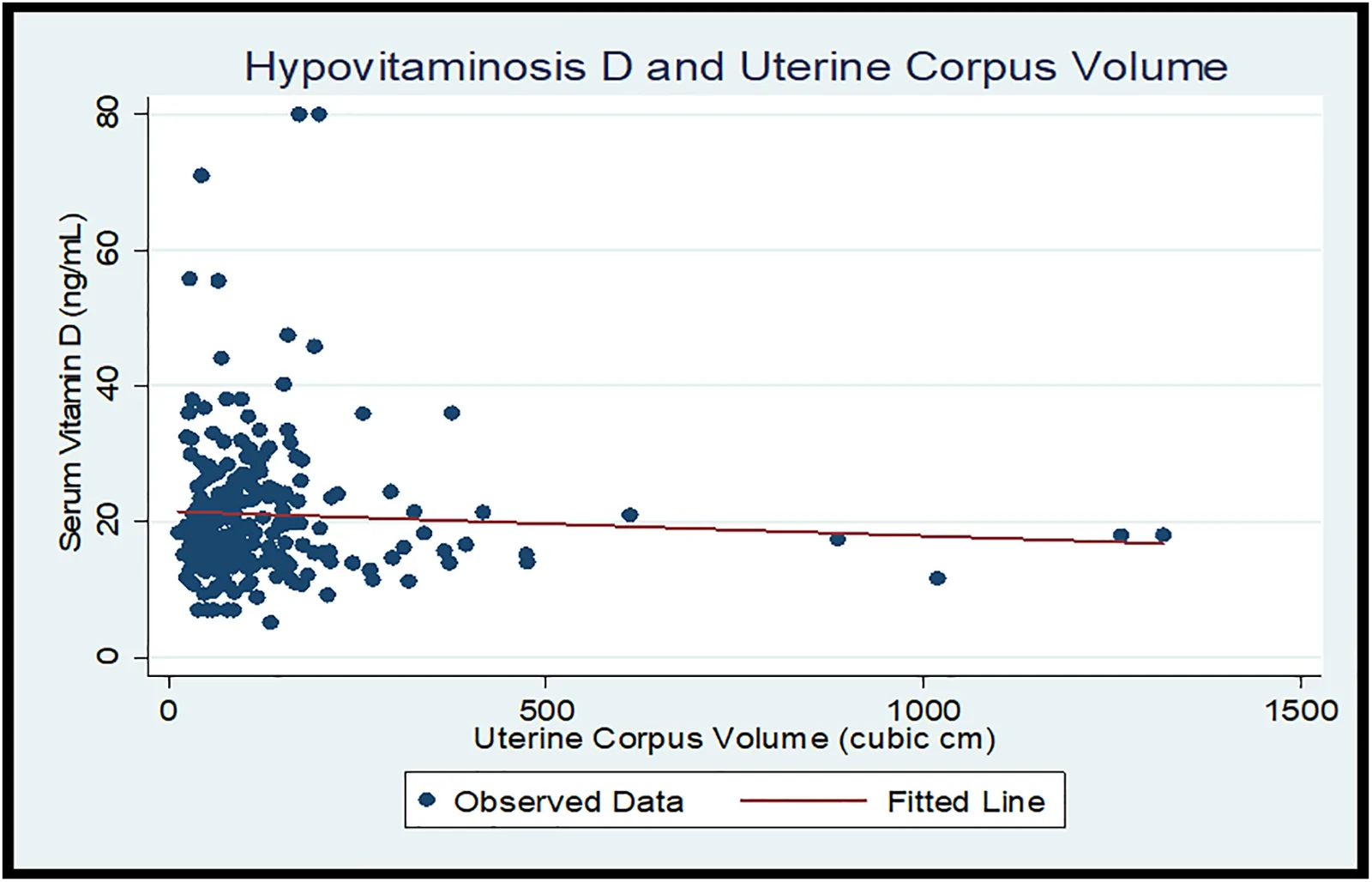
Scatter plot showing the relationship between serum vitamin D levels and uterine corpus volume, with least-squares regression line and 95% confidence intervals.

The Pearson’s correlation coefficients and associated p-values for the relationship between vitamin D levels and various characteristics of uterine leiomyomas was determined. The results indicated a weak negative correlation between the number of leiomyomas and vitamin D levels, but this correlation was not statistically significant (r = − 0.1190, p = 0.3160). The number of leiomyoma sites, however, had statistically significant weak negative correlation with vitamin D levels (r = − 0.2482, p = 0.0342). There was no significant correlation between vitamin D levels and the largest leiomyoma volume or corpus volume. In general, the findings indicated a potential relationship between vitamin D levels and the number of leiomyoma sites (Table 5, Figs 3–6).

**Table 5:** Relationship between vitamin D levels and uterine leiomyoma characteristics.

Variable	Pearson’s correlation coefficient (r)	p-value
Leiomyoma number	−0.1190	0.3160
Leiomyoma site number	−0.2482	0.0342*
Largest leiomyoma volume	−0.0753	0.5265
Corpus volume	−0.0507	0.6701

Figs 3−6 present scatter plots illustrating the relationship between serum vitamin D levels and uterine leiomyoma characteristics. Each point represents an individual participant, while the fitted line denotes the least-squares linear regression with 95% confidence intervals. These figures demonstrate the distribution of vitamin D levels across leiomyoma characteristics and support the Pearson correlation analyses reported in Table 5.

## Discussion

### Prevalence of hypovitaminosis D

The prevalence of hypovitaminosis D was 54.1%. Several studies have been conducted in Africa to assess vitamin D levels in various populations a few however, have assessed this prevalence among women of reproductive age. Similar to this research, the prevalence of hypovitaminosis D among Egyptian women of childbearing age was 56% (43% for Vitamin D deficiency and 13% for Vitamin D insufficiency) [3]. Higher prevalence has been observed among women in Sudan where majority of the women use covered clothing style [4]. Evidence from Ethiopia also indicates substantial vitamin D inadequacy among women. In southern Ethiopia, Gebreegziabher and Stoecker (2013) found that 55.1% of women had serum 25-hydroxyvitamin D concentrations below 40 nmol/L, while only 15.8% had concentrations above 50 nmol/L [27]. More recently, Haile et al. (2022) reported vitamin D deficiency in 39.0% of pregnant women attending antenatal care in Sodo town [28]. Research conducted in India found a prevalence of 88% among women of childbearing age [29]. According to this research majority were from middle socioeconomic class compared to subjects from upper, upper middle, and lower socioeconomic class. The study participants were of the same age group and majority of the participants were rural residents. The higher prevalence of hypovitaminosis D compared to this study may be attributed to variations in culture, geographical location, lifestyle or diet.

The prevalence of Vitamin D deficiency in Uganda is estimated to be between 9–20%, which is lower than the findings of this study. This prevalence was based on two studies one involving male participants with HIV and tuberculosis coinfection and another one involving both male and female elderly participants with acute stroke [5,6]. Participants with hypovitaminosis D were more likely to be from the Basoga ethnic group. This suggests that there may be regional and ethnic differences in the prevalence of vitamin D deficiency in Uganda. The disparities may be attributed to variations in lifestyle and diet. Interestingly, the study found that the percentages of individuals with and without hypovitaminosis D were similar across all age categories, marital status, occupation, education level, religious affiliation, monthly income and alcohol consumption. These findings are similar to a study done by El-Khateeb et al who found a high prevalence of vitamin D deficiency among healthy Jordanian women of reproductive age, with no significant differences observed in the levels of deficiency based on income or alcohol use [30].

Another study on “Vitamin D status and its predictors among women of reproductive age in an urban centre in Nigeria” by Brian-D Adinma et al found a high prevalence of vitamin D deficiency among women of reproductive age in Nigeria, with no significant differences observed in the levels of deficiency based on income or alcohol use [31]. Overall, these studies suggested that income and alcohol use may not be significant factors associated with hypovitaminosis D in women of reproductive age, and that other factors may be more important in contributing to this deficiency.

### Comparing proportions of uterine leiomyomas in relation to hypovitaminosis D

The difference in the frequencies of leiomyomas between individuals with and without hypovitaminosis D in this study was not statistically significant suggesting that hypovitaminosis D did not have a significant association with the presence of leiomyomas in the sample population.

The findings are similar to a study conducted in Democratic Republic of Congo by Ingala et al., 2016 who found no association between hypovitaminosis D and uterine leiomyomas when IOM criteria was used [8]. A meta-analysis by Mohammadi et al., 2020, yielded similar results as they observed that although the level of vitamin D was lower among participants with uterine leiomyomas compared to those without, this difference was only statistically significant among the European and Asian women and not among the African population which included two studies one from Egypt and the other from the Democratic Republic of Congo [8,9,21].

These results were inconsistent with a recent analytical cross-sectional study conducted in Nigeria by Tunau et al whose findings indicated a significant difference when the proportions were compared [10]. Inconsistent results from another study by Singh et al showed that serum levels of 25-hydroxyvitamin D3 were significantly lower in women with leiomyoma than in controls [32].

### Relationship between hypovitaminosis D and uterine leiomyomas and its characteristics

The mean serum vitamin D levels were lower in individuals with uterine leiomyomas compared to those without. However, the difference was not statistically significant. This study finding was similar to a study conducted in Democratic republic of Congo by Ingala et al who found no association between hypovitaminosis D and uterine leiomyomas when IOM criteria was used. However, association was elicited using the Congo local laboratory cutoff criteria of vitamin D deficiency as levels less than <4ng/mL [8].

Another similar finding is found from research done in America by Mitro et al., 2015. In their study on 3600 women who took part in the National Health and Nutrition Examination Survey (NHANES) between 2001 and 2006, they found no association between low vitamin D levels and the appearance of uterine leiomyomas within the entire population [33].

In contrast to the outcomes of this study, recent research by Guo et al. and Tunau et al indicates a potential causal relationship between hypovitaminosis D and uterine leiomyomas. Guo et al. conducted a Mendelian randomization study, which supported a causal association between genetically predicted vitamin D levels and the susceptibility to uterine leiomyomas. Similarly, Tunau et al. identified a significant association between hypovitaminosis D and leiomyomas in Nigeria, noting lower average vitamin D levels among cases in comparison to controls. Furthermore, studies by All ansary et al, Baird et al and Ciebiera et al echoed these findings, suggesting an increased likelihood of vitamin D deficiency or specific vitamin D receptor gene polymorphisms among women with leiomyomas [7,10,25,34,35].

In relation to uterine leiomyoma characteristics, this study found a negative association between vitamin D levels and various factors such as leiomyoma number, site number, volume of the largest leiomyoma, and corpus volume. Particularly noteworthy was the statistically significant weak negative correlation between vitamin D levels and leiomyoma site number, though the correlation with leiomyoma number did not attain statistical significance. Nonetheless, these correlations, while negative, were deemed inconsequential concerning corpus and leiomyoma volumes. These results align with those of Paffoni et al, which similarly noted a correlation between vitamin D insufficiency and the number, rather than the size, of uterine leiomyomas [22].

Furthermore, corroborating evidence comes from studies such as the randomized clinical trial by Arjeh et al, where no substantial reduction in leiomyoma size was observed among those receiving vitamin D supplementation compared to the placebo group [36]. Similarly, Singh et al. (2019) found no significant relationship between serum vitamin D levels and either the number of uterine leiomyomas or the volume of the largest leiomyoma [32], A weak inverse correlation was observed between serum vitamin D level and leiomyoma site number. However, this finding should be interpreted cautiously because the effect size was small, multiple leiomyoma characteristics were examined, and the corresponding categorical comparison for more than one site was not statistically significant. The observed correlation may therefore represent a chance finding rather than a clinically meaningful association.

In India, Ajmani et al conducted research that revealed a significant negative correlation between vitamin D levels and the volume of uterine leiomyomas [24], while Sabry et al. (2013) found a statistically significant inverse correlation between serum vitamin D levels and total uterine leiomyoma volume [21]. Similarly, Hajhashemi et al conducted a randomized, double-blind, placebo-controlled trial among 69 vitamin D-deficient premenopausal women with uterine leiomyomas. After 10 weeks, women receiving vitamin D_3_ supplementation had significantly smaller mean leiomyoma diameters than those receiving placebo, although the small sample size and short follow-up period limit definitive conclusions [37]. However, these findings contrast with those of our research, as the vitamin D levels among their cases were notably lower compared to the leiomyoma cohort in our study. The differing study designs may account for these inconsistencies. Nonetheless, it is plausible that factors other than hypovitaminosis D contribute to the development of leiomyoma among our study participants [24].

The significant difference in vitamin D levels between women with and without uterine leiomyomas, particularly among individuals of European and Asian descent, suggests several potential factors at play. Reduced sunlight exposure, a primary source of vitamin D, could be a contributing factor, possibly stemming from decreased outdoor activity or heightened sunscreen use. Furthermore, dietary habits may play a role, with women affected by leiomyomas potentially having lower vitamin D intake due to dietary variations or factors hindering nutrient absorption. Hormonal imbalances, such as estrogen dominance, might impact vitamin D metabolism, as estrogen has been linked to increased production of vitamin D binding protein. Moreover, the association between leiomyomas and chronic inflammation could further disrupt vitamin D metabolism. Finally, genetic predispositions may also influence vitamin D metabolism, potentially contributing to the observed differences in vitamin D levels among affected individuals [7,23,38–40].

Unlike in other populations, the variation in vitamin D levels among Africans did not show statistical significance. This could stem from genetic disparities, as research indicates that individuals of African heritage might metabolize vitamin D differently. Moreover, dietary habits may play a role, with some studies proposing that individuals of African descent consume more vitamin D-rich foods, potentially leading to higher blood levels of the vitamin. Environmental factors, including sunlight exposure and pollution, may also influence vitamin D levels among African populations [34,41,42].

While the study did not find significant associations between leiomyoma occurrence and majority of its characteristics examined in relation to hypovitaminosis D, there was negative weak correlation between more than one leiomyoma site and vitamin D levels which was statistically significant and this warrants further investigation. The negative correlation noted between serum vitamin D levels and uterine leiomyoma number, largest leiomyoma volume and corpus volume was weak and not statistically significant. Similar research studies have yielded mixed results, and more research is needed to better understand the relationship between vitamin D levels and leiomyomas.

### Study strengths and limitations

This study is, to our knowledge, the first to report the prevalence of hypovitaminosis D among women of reproductive age in Uganda and the first to examine its relationship with uterine leiomyoma and selected sonographic characteristics in this population. The multicenter design, involving three regional referral hospitals, improved the geographic diversity of the sample.

However, several limitations should be considered. The cross-sectional design precludes causal inference and does not allow determination of whether hypovitaminosis D preceded uterine leiomyoma occurrence. Although the study was multicenter, participants were recruited from only three regional referral hospitals and may not be representative of all women of reproductive age in Uganda. In addition, the sample size may have limited power to detect modest associations, particularly because only 73 participants had uterine leiomyoma. The findings should therefore be interpreted cautiously.

## Conclusion and recommendations

Hypovitaminosis D was common among women of reproductive age attending the participating hospitals in Uganda. However, after adjustment for potential confounders, this study did not demonstrate a statistically significant independent association between hypovitaminosis D and uterine leiomyoma. Although a weak inverse correlation was observed between serum vitamin D levels and leiomyoma site number, this finding should be interpreted cautiously.

The high prevalence of hypovitaminosis D observed in this study highlights its public health importance among women of reproductive age in Uganda. Healthcare providers should strengthen health education on prevention of vitamin D deficiency through appropriate sunlight exposure and dietary improvement. Further large, analytically robust studies with adequate adjustment for confounding are needed to determine whether vitamin D status is independently associated with uterine leiomyoma occurrence or specific sonographic characteristics

## Supporting information

S1 Data Anonymized dataset used for the analysis of the association between hypovitaminosis D and uterine leiomyomas among women of reproductive age attending selected hospitals in Uganda. (XLSX)

## Abbreviations

FIGO: International Federation of Gynecology and Obstetrics
IOM: Institute of medicine
cOR: crude Odds ratio
CI: Confidence Interval
N: total number
n: number
Ref: reference
